# Primary Extrahepatic Bile Duct Carcinoids

**DOI:** 10.1155/1996/45231

**Published:** 1996

**Authors:** Giulio Belli, Gianluca Rotondano, Alberto D'Agostino, Ines Marano

**Affiliations:** 1 Department of General Surgery & Organ Transplantation University of Naples "Federico II" School of Medicine Naples Italy; 2 Dept. of Radiology I University of Naples "Federico II" School of Medicine Naples Italy

## Abstract

Biliary tact carcinoids are extremely rare: only ten cases have been reported up to now. The Authors describe a successfully treated carcinoid tumour ofthe proximal bile duct and review the literature about these rare malignancies.

Despite extensive preoperative work-up, including ultrasound, CT scan and ERCP, a definite diagnosis is hardly possible prior to histologic examination of the operative or necropsy specimen.

Due to the slow-growing nature and the non-aggressive behaviour of these malignancies, surgical resection followed by biliodigestive anastomosis should be the treatment of choice.


*HPB Surgery*, 1996, Vol. 9, pp. 101–105
Reprints available directly from the publisher
Photocopying permitted by license only

© 1996 OPA (Overseas Publishers Association)
Amsterdam B.V. Published in The Netherlands
by Harwood Academic Publishers GmbH
Printed in Malaysia

### CASE REPORT

### Primary Extrahepatic Bile Duct Carcinoids

GIULIO BELLI, GIANLUCA ROTONDANO, ALBERTO D'AGOSTINO and INES MARANO*

Department of General Surgery & Organ Transplantation and *Dept. of Radiology I, University of Naples "Federico II" School of Medicine, Naples, Italy

(*Received October 23, 1993*)

Biliary tact carcinoids are extremely rare: only ten cases have been reported up to now. The Authors describe a successfully treated carcinoid tumour of the proximal bile duct and review the literature about these rare malignancies.

Despite extensive preoperative work-up, including ultrasound, CT scan and ERCP, a definite diagnosis is hardly possible prior to histologic examination of the operative or necropsy specimen.

Due to the slow-growing nature and the non-aggressive behaviour of these malignancies, surgical resection followed by biliodigestive anastomosis should be the treatment of choice.

KEY WORDS: Biliary tract carcinoid tumours surgery.

### INTRODUCTION

Carcinoid tumours (argentaffinoma) may occur anywhere in the distribution of the argentaffin cell system. They are derived from Kultschizky cells and have the potential to produce serotonin. Cases occurring in the bladder, prostate, rectum, stomach, bronchi, pancreas and biliary tract have been reported, in addition to the more common sites in the ileum and appendix[1].

Biliary tract carcinoids are exceedingly rare. Jutte and coworkers in 1986 surveyed the world literature and found 23 cases, seventeen arising in the gallbladder and seven in the extrahepatic bile ducts[2]. Since then there appears to have been only two further reported cases, to our knowledge[3,4].

In this paper we describe a successfully treated carcinoid tumour of the proximal common bile duct and review the literature about these rare malignancies.

*Address for correspondence:* Giulio Belli, Via Cimarosa 2A, 80127, Naples, Italy. Phone and fax: 081/7462550.

### CASE-REPORT

A 78-years old caucasian male was referred to our institution for obstructive jaundice and itching. Physical examination revealed a well-nourished man with stable vital signs. His liver was palpated 1 cm below the right costal margin and no palpable mass or abdominal tenderness was noted. White blood cell count was 6.1 cells/mmc, hemoglobin concentration was 13.2 g/dl. Serum electrolytes and coagulation tests were normal. Total bilirubin was 14.6 mg/dl, alkaline phosphatase 376 U/l, LDH 324 U/l, gammaglutamyl transpeptidases 248 U/l, AST 155 U/l and serum amylase 113 U/l (upper limit of normal 220 U/l). Urinalysis was normal. Radioimmunologic tests were negative for anti-HBs and anti-HCV antibodies as well as for CEA and alpha-fetoprotein.

Chest x-rays was normal. Abdominal ultrasound revealed a dilated biliary tract. No gallstones were seen. CT scan confirmed marked dilatation of intrahepatic bile ducts but was not able to detect any mass in the region of the porta hepatis. Arteriography indicated a narrowing and displacement of the gastroduo-

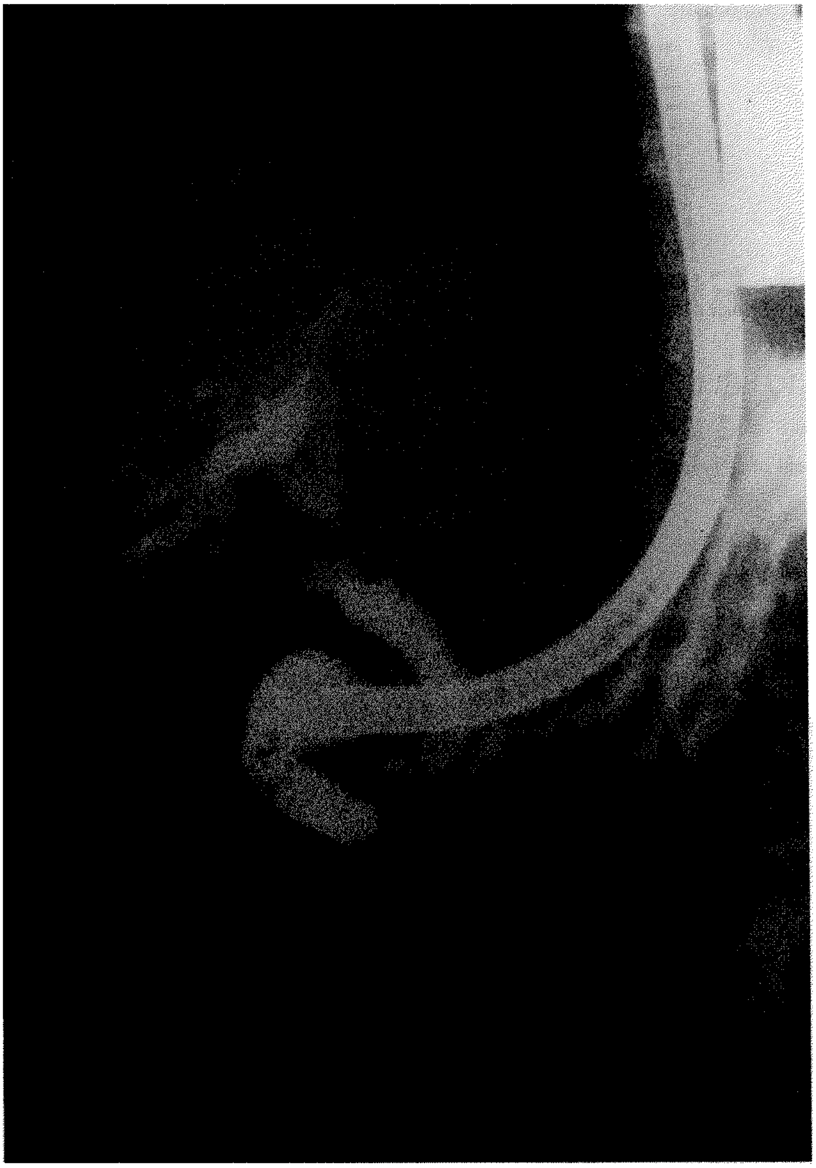

**Figure 1** ERCP showing a stricture of the proximal bile duct and the dilatation of intrahepatic biliary tree.

denal artery, with a focus of hypervascularization at the hilum of the liver. The angiographic examination also revealed the right hepatic artery arising from the superior mesenteric artery.

Finally, an endoscopic retrograde cholangiopancreatography (ERCP) showed a 1.5 cm long narrowing of the proximal common bile duct up to the junction of the left and right hepatic ducts. Intrahepatic biliary tree was markedly dilated (Figure 1).

Laparotomy was performed through a right subcostal incision. The gallbladder was removed. A 1.5 rubber-like mass was encasing the common bile duct. Biliary system was divided immediately above the duodenum and its lower part was dissected from the underlying portal vein and hepatic artery together with the associated connective tissue and lymphnodes. Then the biliary duct was transected at the hilum junction, the specimen removed and a Roux-en-Y hepatico-Jejunostomy performed.

*Postoperative course was uneventful:* bilirubin levels fell to 7.7 mg/dl on the fourth postoperative day and the patient was discharged on the twelveth postoperative day.

The patient remains well 15 months after surgery with normal bilirubin levels (0.9 mg/dl) and no clinical or radiological signs of recurrent disease or meta-

**Figure 2** Histologic examination: alveolar infiltration from a well-differentiated malignancy. Intense cytoplasmatic positive reaction to chromogranine (PAP 40 X). See Color Plate I.

stases. An hepatobiliary HIDA scintigraphic scan confirmed a good function of the bilio-digestive anastomosis and an abdominal ultrasound revealed a normal aspect of the liver and of the intrahepatic biliary tree.

*Pathology:* the neoplasm was 1.5 × 0.8 × 0.6 cm rubber-like mass showing focal areas of necrosis and calcification. The tumor consisted of small pleiomorphic cells, arranged either in solid nests or in alveolar clusters. Cytoplasm was eosinophilic with centrally located nuclei. Occasional mitotic figures were seen. Immunohistochemical examination with PAP and chromogranine stain gave intensely positive reaction (Figure 2). Gallbladder and periductal lymphnodes were free of tumour. Final diagnosis was *carcinoid tumour of the common bile duct.*

### DISCUSSION

Carcinoid tumours are found in 1.2% of all autopsies[1,5]. Biliary tract carninoids represent 0.2–2% of digestive carcinoids[6,7].

Although originally considered to be benign lesions, most pathologists would now agree that all carcinoids, or certainly all extraappendiceal ones, are potentially malignant. The usual criteria of cell anaplasia and mitoses are not applicable and definitive evidence of malignancy must rely on evidence of gross or microscopic invasiveness[2].

While the jejunoileum, appendix and rectum are relatively frequent sites for carcinoids, the biliary system is an extremely rare site because of the low number of enterochromaffin cells located in this area. Pilz was the first to describe a case of carcinoid tumour of the biliary duct in 1961[8] and, up to date, to our knowledge, eleven cases of extrahepatic biliary duct carcinoids, including the present one, have been reported (Table 1).

In support of the primary nature of the tumour are the operative findings (no other tumour masses found on complete abdominal exploration) and histological features of the specimen: the architectural pattern of the neoplasm differs considerably from the packeted cells arrangement normally associated with ileal or appendiceal carcinoids. Instead, the tumour is

**Table 1** Carcinoid tumors of extrahepatic bile ducts

| Case | Author | Year | Sex | Age | Symptoms | Diagnosis | Metastases | Treatment | Outcome |
|---|---|---|---|---|---|---|---|---|---|
| 1 | Pilz[8] | 1961 | F | 22 | – | At operation | None | Biopsy | – |
| 2 | Little[9] | 1968 | F | 41 | Jaundice Weight loss | At operation | Portal vein invasion | Biopsy | Died 3 weeks postop. |
| 3 | Godwin[6] | 1975 | – | – | – | At operation | Liver | – | Died 1 day postop. |
| 4 | Berghdal[10] | 1976 | F | 79 | None | Autopsy | None | – | – |
| 5 | Judge[11] | 1976 | M | 19 | Pain, nausea, vomiting | Autopsy | None | – | – |
| 6 | Gerlock[12] | 1979 | M | 32 | Jaundice | At operation | None | – | – |
| 7 | Vitaux[13] | 1981 | M | 24 | Jaundice | At operation | Pancreas | Pancreatico-duodenectomy | Alive 24 ms postop. |
| 8 | Jutte[2] | 1986 | M | 62 | Back pain | At operation | None | Resection of tumor, Roux-en-Y hepaticojejunostomy | Alive 25 ms postop. |
| 9 | Bickerstaff[3] | 1987 | F | 57 | Jaundice | At operation | None | – | Alive 6 ms postop. |
| 10 | Bumin[4] | 1990 | F | 38 | Jaundice back pain | At opration | None | Mass removal T-tube | – |
| 11 | Belli | 1993 | M | 78 | Jaundice | At operation | None | Tumor resection Roux-en-Y hepaticojejunostomy | Alive 15 ms postop. |

ms = months arranged with characteristic cytoplasmic granulation of enterochromaffin cells.

The mean age of patients was 42 (range 17–79). In nine patients whose sex was known, there were 5 males and 4 females, with obvious differences from cholangiocarcinomas in which the mean age is 65 and the male/female ratio is 3:1[4]. The presenting symptoms were abdominal or back pain, weight loss, jaundice, nausea and vomiting or pain from metastases.

A confident diagnosis of carcinoid tumour cannot be made preoperatively, and most often the disorder is misdiagnosed as choledocolithiasis or cholangiocarcinoma. In fact, the diagnosis was made at operation in nine cases and at necropsy in two.

Since jaundice is the commonest presenting symptoms, ERCP is considered the best approach for diagnosis. The usefulness of ERCP is demonstrated by our case where it showed a 1.5 cm long stricture in the upper common bile duct, clearly defining the level of the lesion and the dilatation of the intrahepatic ducts.

Nonetheless, it is very hard to tell between a retropancreatic bile duct malignancy and a pancreatic cancer encasing the bile duct[12]. Abdominal ultrasound has also been employed and usually demonstrates bile duct dilatation. These findings, together with a normal appearance of the pancreatic head, would favor a diagnosis of extrahepatic bile duct cancer, although a small pancreatic cancer or ampullary neoplasm should first be excluded[14].

CT scan and percutaneous transhepatic cholangiography (PTC) may well be helpful in detecting the lesion, the latter being able to show the proximal level of the stricture thus giving further information about the nature of the disease, but a precise diagnosis is very unlikely to be made prior to histologic examination of an operative or necropsy specimen.

It has been estimated that 6–10% of patients with carcinoids had serotonin overproduction and carcinoid syndrome[15]. None of the cases of biliary tract carcinoids in the literature was associated with the syndrome. The absence of clinical features of the carcinoid syndrome in the present report is not surprising in view of the localized nature of the malignancy and absence of liver metastases at operation.

The prognosis of these neoplasms is hard to assess, since the cases are rare and little information is available about patients' follow-up. By the way, biliary tract carcinoids appear to be non-aggressive tumours, since a 20-years survival without operation has been reported[9].

Surgical excision of the tumour is probably the treatment of choice. Only two of the ten previously reported bile duct carcinoids were resected, one by pancreaticoduodenectomy[13] and one by resection of the common bile duct with a Roux-en-Y hepaticojejunostomy[2]. Our patient underwent a common hepatic duct resection followed by a Roux-en-Y hepatico-jejunostomy at the hilum junction, due to the high biliary location of the tumour.

As for adjuvant treatment, radiotherapy is generally considered of little benefit in the management of carcinoids, whereas chemotherapy with 5-FU has proved to be of some palliative value in carcinoids of other sites[2].

Considering the non-aggressive behaviour and the slow-growing nature of these malignancies, major surgery is reasonable and fully justified, whenever possible, particularly in fit patients. Biliary continuity should then be restored by biliodigestive anastomoses.

### REFERENCES

1. Thompson, G. B., Van Herdeen, J. A., Martin, J. K., Schutt, A. J., Ilstrup, D. M., Carrey, J. A. (1985) "Carcinoid tumours of the gastrointestinal tract: presentation, management and prognosis". *Surgery,* **98**, 1054–1063.
2. Jutte, D. L., Bell, R. H., Penn, I., Powers, J., Kolinjivadi, J. (1986) "Carcinoid tumour of the biliary system". *Dig. Dis. Sci.,* **32**, 763–769.
3. Bickerstaff, D. R., Ross, W. B. (1987) "Carcinoid of the biliary tree". *Journal of the Royal College of Surgeons of Edimburgh,* **32**, 48–51.
4. Bumin, C., Ormeci, N., Dolapci, M., Gungor, S. (1990) "Carcinoid tumour of the biliary duct". *Int. Surg.,* **75**, 262–264.
5. Martensson, H., Nobin, A., Sundler, F. (1983) "Carcinoid tumours in the gastrointestinal tract. An analysis of 156 cases". *Acta. Chir. Scand.,* **149**, 607–616.
6. Godwin, J. D. (1975) "Carcinoid tumours, an analysis of 2837 cases". *Cancer,* **36**, 560–569.
7. Welch, J. P., Malt, R. A. (1977) "Management of carcinoid tumours of the gastrointestinal tract". *Surg. Gynecol. Obstet.,* **145**, 223–227.
8. Pilz, E. (1961) "Uber ein karzinoid des ductus choledocus". *Zbl. Chir,* **86**, 1588–1590.
9. Little, J., Gibson, A. A. M., Kay, A. W. (1968) "Primary common bile duct carcinoid". *Br. J. Surg.,* **55**, 147–149.
10. Berghdal, L. (1976) "Carcinoid tumours of the biliary tract". *Aust. Nz. J. Surg.,* **46**, 136–138.
11. Judge, D. M., Dickman, P. S., Trapuki, B. S. (1976) "Non-functioning argyrophilic tumor (apudoma) of the hepatic duct". *Am. J. Clin. Pathol.,* **66**, 40–45.
12. Gerlock, A. J., Muhletaler, C. A. (1979) "Primary common bile duct carcinoid". *Gastrointestinal Radiology,* **4**, 263–264.
13. Vitaux, J., Salmon, R. J., Langville, O., Buffet, C., Martin, E., Chaput, J. C. (1981) "Carcinoid tumour of the common bile duct". *Am. J. Gastroenterol,* **76**, 360–362.
14. Levine, E., Makland, N. F., Wright, C. H. *et al.* (1979) "Computed tomography and ultrasound appearance of primary carcinoma of the common bile duct". *Gastrointestinal Radiology,* **4**, 147–151.
15. Feldman, J. M. (1987) "Carcinoid tumour and syndrome". *Semin. Oncol.,* **14**, 237–246.

